# Ascorbic acid supplementation in type 2 diabetes mellitus: A protocol for systematic review and meta-analysis

**DOI:** 10.1097/MD.0000000000023125

**Published:** 2020-11-06

**Authors:** Lipeng Shi, Xuqin Du, Pei Guo, Lumei Huang, Peng Qi, Qianhui Gong

**Affiliations:** a Clinical Department, Dianjiang Hospital of Traditional Chinese Medicine, Dianjiang, Chongqing; b College of Traditional Chinese Medicine, Chongqing Medical University; c Hospital of Chengdu University of Traditional Chinese Medicine, Chengdu, Sichuan Province; d Graduate School, Hunan University of Traditional Chinese Medicine, Changsha, Hunan Province, China.

**Keywords:** ascorbic acid supplementation, glycemic control, insulin resistance, meta-analysis, protocol, systematic review, type 2 diabetes mellitus

## Abstract

**Background:**

Diabetes is one of the most common chronic diseases in the world. In recent years, with the continuous improvement of people's living standards and changes in dietary structure, the incidence of diabetes is gradually increasing. Studies have shown that ascorbic acid supplementation can reduce blood glucose, increase insulin synthesis and secretion, improve insulin resistance, and reduce the occurrence and development of complications of type 2 diabetes mellitus (T2DM). However, relevant studies have common problems such as the lack of large sample studies and low quality of included studies. Therefore, it is needed that we meta-analyze the clinical trials with high quality to elucidate the efficacy and safety of ascorbic acid supplementation in patients with T2DM.

**Methods:**

We will search randomized controlled trials published by PubMed, Embase, the Cochrane Library, Web of Science, and the Clinical Trials.gov website from inception to August 2020 on the effects of ascorbic acid supplementation on blood glucose, glycosylated hemoglobin, serum insulin, insulin resistance and other variables in T2DM patients with no language restrictions. The retrieval adopts the combination of medical subject headings and random words, and traces the references of the included literature to supplement the acquisition of relevant literature. Two researchers will independently screen the retrieved literature, extract the data and cross-check, and the Review Manage software V5.3.0 will be utilized for meta-analysis.

**Results:**

Our study will provide a high-quality and in-depth comprehensive analysis of the effects of ascorbic acid supplementation on blood glucose control, glycosylated hemoglobin and insulin resistance in type 2 diabetic patients.

**Conclusion:**

This systematic review and meta-analysis concerning randomized controlled trials of ascorbic acid supplementation for type 2 diabetic patients will provide a new direction and strong evidence to evaluate whether ascorbic acid supplementation is of benefit to glucose control and insulin resistance in T2DM.

**PROSPERO registration number:**

CRD 42019146826

## 1 Introduction

Type 2 diabetes mellitus (T2DM) is a chronic non-communicable disease characterized by early insulin resistance, late islet β-cell failure and hyperglycemia, accounting for 90% of diabetic patients.^[1]^ In 2015, 415 million people aged 20 to 79 years suffered from DM globally, 5.0 million deaths were attributed to diabetes, and it has been predicted to reach 642 million people aged 20 to 79 years in 2040.^[2]^ Diabetes is reported to be an independent hazard factor that may cause cardiovascular disease (CVD). Compared with non-diabetic patients, the risk of diabetic patients who suffer from CVD increases by 2 to be 4-fold. Abnormal glucose and lipid metabolism play a critical role in the occurrence and development of CVD in patients with T2DM.^[3,4]^

Various studies have established that oxidative stress in diabetic patients can lead to the occurrence and development of diabetes and its complications.^[5–7]^ Oxidative stress reflects an imbalance of oxidation and anti-oxidation in the body, and the production of free radicals in the tissues exceeds the scavenging ability of the endogenous anti-oxidation defense system, thus leading to the injury of the body's tissues and cells, resulting in cell dysfunction or death.^[8]^ Studies have shown that long-term hyperglycemia mainly leads to the reactive oxygen species generation through glycosylation, glucose oxidation and polyols, which damages β-cells and leads to impaired insulin release and insulin resistance.^[9,10]^ On the other hand, people with a low concentration of anti-oxidants are more likely to raise blood glucose, increase insulin resistance and increase the risk of various diabetes complications.^[11]^ Studies have demonstrated that oxidative stress caused by excessive free radicals in the body is closely related to insulin resistance and islet βcell dysfunction, and is an important factor leading to the occurrence and development of diabetes.^[12,13]^ Therefore, anti-oxidation supplementation by enhancing antioxidant defenses can improve insulin resistance and improve β-cell function.^[9]^

Ascorbic acid, also known as Vitamin C, is an effective water-soluble antioxidant, which has a scavenging effect on excessive free radicals in the body of diabetic patients and a protective effect on tissue damage caused by oxidative stress.^[14,15]^ Some studies have found that ascorbic acid supplementation can improve islet cell function in patients with T2DM, which can be used for the early prevention of diabetes and the later treatment of complications.^[16,17]^ Furthermore, some studies,^[18,19]^ but not others,^[20]^ have shown that ascorbic acid supplementation can regulate fasting blood glucose (FBG), glycosylated hemoglobin (HbA1c) and improve insulin resistance.

Overall, the supplementation of ascorbic acid is closely related to the improvement of glycemic level and insulin sensitivity in diabetics, although there is still controversy.

This article will systematically evaluate the effect of ascorbic acid supplementation on FBG, HbA1c, and insulin resistance in patients with T2DM, and total cholesterol, triglyceride, high density lipoprotein, low density lipoprotein, adverse events will be evaluated when necessary.

## 2 Methods

### 2.1 PROSPERO registration

The protocol of this study has been registered on PROSPERO platform (No: CRD 42019146826).

### 2.2 Eligibility criteria

#### 2.2.1 Types of trials

This meta-analysis will include randomized controlled trials (RCTs) of ascorbic acid supplementation in patients with T2DM. Clinical observation, cohort studies, case-control, and laboratory studies will be excluded. And there are no language restrictions.

#### 2.2.2 Types of patients

Adult patients diagnosed with T2DM will be included in the meta-analysis. The diagnostic criteria of diabetes are in accordance with World Health Organization or American Diabetes Association criteria. And there are no gender or race restrictions.

#### 2.2.3 Types of interventions

RCTs of ascorbic acid supplementation combined with conventional diabetic medication for T2DM will be included, with administration lasting at least 4 weeks. And there are no restrictions on the frequency and dosage administration.

#### 2.2.4 Types of controls

RCTs of placebo supplementation combined with conventional diabetic medication for T2DM will be included. Moreover, conventional medication for diabetes remains consistent between the 2 groups.

#### 2.2.5 Types of outcome measurements

##### 2.2.5.1 Primary outcomes

The primary outcome indicators of the meta-analysis include FBG, HbA1c, serum insulin, and insulin resistance.

##### 2.2.5.2 Secondary outcomes

The secondary outcome indicators of this study are total cholesterol, triglyceride, high density lipoprotein, low density lipoprotein, and adverse events.

### 2.3 Search methods for the identification of eligible trials

We will search RCTs published by PubMed, Embase, the Cochrane Library, Web of Science, and the Clinical Trials.gov website from inception to August 2020 on the effects of ascorbic acid supplementation on blood glucose, HbA1c, serum insulin, insulin resistance and other variables in T2DM patients. The retrieval adopts the combination of medical subject headings and random words, and traces the references of the included literature to supplement the acquisition of relevant literature. The search terms are as follows, including: “ascorbic acid,” “vitamin C,” “*vit.* C,” “diabetes mellitus,” “type 2 diabetes mellitus,” “type 2 diabetes,” “type II,” “randomized controlled trial,” “randomised controlled trial,” “controlled clinical trial,” “clinical trial,” “randomized,” “randomised, trial.” Take PubMed as an example, its retrieval strategy is listed in Table 1.

**Table 1:** PubMed search strategy.

#1 ascorbic acid
#2 vitamin C
#3 vit. C
#4 #1 OR #2 OR #3
#5 diabetes mellitus
#6 type 2 diabetes mellitus
#7 type 2 diabetes
#8 type II
#9 #5 OR #6 OR #7 OR #8
#10 randomized controlled trial
#11 randomised controlled trial
#12 controlled clinical trial
#13 clinical trial
#14 randomized
#15 randomised
#16 trial
#17 #10 OR #11 OR #12 OR #13 OR #14 OR #15 OR #16
#18 #4 AND #9 AND #17

### 2.4 Study selection

**Figure 1:**
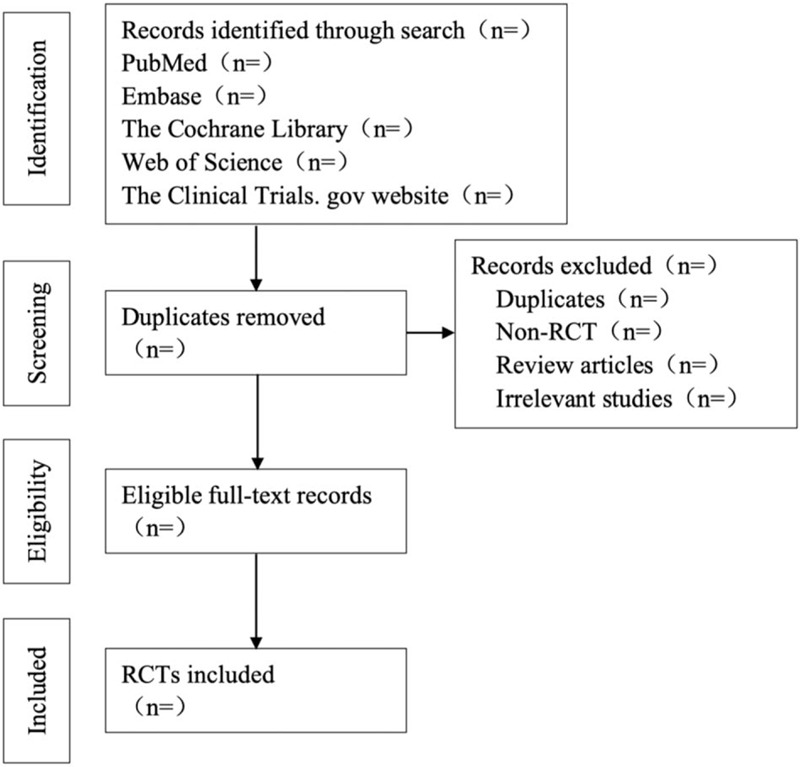
Flow chart of the study selection.

Two researchers will screen the retrieved literature according to inclusion and exclusion criteria. If there is any difference, it will be discussed and resolved or the third reviewer will assist in the judgment. For the lack of documents, we will try our best to contact the original author to supplement the missing literature. And we will import the literature retrieved from the database into Endnote software, and duplicate literature will be deleted. This study will employ the Preferred Reporting Items for Systematic Reviews and Meta-Analyses recommended methods and checklists to report our findings.^[21]^ Besides, the flow chart (Fig. 1) will be utilized for literature identification and screening.

### 2.5 Data extraction and management

According to the inclusion criteria, 2 reviewers will use the same eligibility assessment form to evaluate the included literature. All included studies will be recorded the following information:

1. Basic information included in the study: including the title, author's name, year of publication, country;
2. Basic characteristics of the study subjects: including age, gender, body mass index, number of each group, sample size, randomized method, allocation concealment, and blind method;
3. Specific details and treatment course of intervention measures;
4. The key elements of bias risk assessment;
5. Focus on the outcome indicators of the main data.

### 2.6 Missing data management

In order to obtain relevant data, we will do our best to get in touch with the author. If the relevant missing data is not available, it will be eliminated. Furthermore, we will analyze the impact of the missing data on the results of this study through sensitivity analysis.

### 2.7 Risk of bias assessment

The bias risk assessment included in the study will be evaluated using the RCT risk assessment tool recommended in Cochrane 5.1.0. Specifically, including:

1. Whether the random method is correct;
2. Whether the distribution is hidden;
3. Whether the subjects and researchers use blind method;
4. Whether the result data is complete;
5. Whether there is a selective report of research results;
6. Other sources of bias.

The risk of bias will be assessed independently by 2 evaluators, and the results will be cross-checked. Any differences will be discussed and resolved or the third reviewer will assist in the judgment.

### 2.8 Statistical analysis

Using the Review Manage software V5.3.0, we will perform a meta-analyze of the literature that meets the inclusion criteria. As all outcome indicators are continuous variables, mean difference or standard mean difference, and 95% confidence interval will be used as effect analysis statistics. The heterogeneity of these included studies will be accurately assessed by the Chi-square test (*χ*^2^) and *I*^2^ statistic. If the heterogeneity test result *I*^2^ < 50%, a fixed effect model will be utilized for meta-analysis. If the heterogeneity test results *I*^2^ ≥ 50%, it indicates that statistical heterogeneity exists between the results of various studies. Also, it is necessary for us to further analyze the origin of heterogeneity. After excluding significant clinical and methodological heterogeneity, a meta-analysis will be performed using a random effect model.

### 2.9 Additional analysis

#### 2.9.1 Subgroup analysis

This study will carry out a subgroup analysis according to the dose and duration of ascorbic acid supplementation.

#### 2.9.2 Sensitivity analysis

For the sake of confirm whether the meta-analysis results are stable, the sensitivity analysis will be performed after the individual studies are sequentially excluded, and to evaluate the difference between the results after elimination and the original merged results.

#### 2.9.3 Reporting bias

In the case of a meta-analysis involving no less than 10 studies, we will use the funnel plot symmetry test for publication bias and carefully interpret the results.

#### 2.9.4 Confidence in cumulative evidence

We will employ the Grading of Recommendations Assessment, Development, and Evaluation profiler 3.2 software to assess the quality of evidence and recommended strength of primary and secondary outcomes. The factors that reduce the quality level include limitations, inconsistencies, whether it is direct evidence, accuracy or confidence interval, and publication bias, each of which reduces by 1 or 2 points according to the specific situation. And effect values, possible confounding factors, and dose-effect relationships are 3 additional factors of increasing evidence grade. The level of evidence quality will be divided into high, moderate, low and very low, with a strong recommendation and weak recommendation.

## 3 Discussion

Diabetes is a chronic disease that threatens human health and is caused by metabolic disorders of the endocrine system that can cause long-term damage to tissues and organs of the cardiovascular, endocrine, neurological, and urinary systems.^[22]^ Many studies have shown that people suffering from impaired glucose tolerance as well as diabetes have been varying degrees of oxidative stress.^[23,24]^ Ascorbic acid, as a natural antioxidant, can directly or indirectly exert an antioxidant role in the body, playing a vital part in improving the level of antioxidants in the body and preventing and treating diabetes. The meta-analysis performed in this article concerning high-quality RCTs of ascorbic acid supplementation in patients with T2DM will provide a new direction and strong evidence for the clinical treatment of T2DM. The purpose of this systematic review and meta-analysis is to evaluate the effects of ascorbic acid supplementation on glycemic control, HbA1c, and insulin resistance in adults with T2DM. In conclusion, we will clearly elucidate the effect and adverse reactions of ascorbic acid supplementation on T2DM.

## Author contributions

**Conceptualization:** Lipeng Shi, Xuqin Du.

**Data curation:** Pei Guo.

**Funding acquisition:** Lumei Huang.

**Investigation:** Lipeng Shi.

**Methodology:** Xuqin Du.

**Project administration:** Xuqin Du.

**Software:** Lipeng Shi.

**Supervision:** Qianhui Gong.

**Validation:** Peng Qi.

**Writing – original draft:** Lipeng Shi, Xuqin Du.

**Writing – review and editing:** Lipeng Shi, Xuqin Du.
